# How Cations Can Assist DNase I in DNA Binding and Hydrolysis

**DOI:** 10.1371/journal.pcbi.1001000

**Published:** 2010-11-18

**Authors:** Marc Guéroult, Daniel Picot, Joséphine Abi-Ghanem, Brigitte Hartmann, Marc Baaden

**Affiliations:** 1 CNRS UPR 9080, Institut de Biologie Physico-Chimique, Paris, France; 2 INTS, INSERM UMR S665, Paris, France; 3 CNRS UMR 7099, Institut de Biologie Physico-Chimique, Paris, France; Stanford University, United States of America

## Abstract

DNase I requires Ca^2+^ and Mg^2+^ for hydrolyzing double-stranded DNA. However, the number and the location of DNase I ion-binding sites remain unclear, as well as the role of these counter-ions. Using molecular dynamics simulations, we show that bovine pancreatic (bp) DNase I contains four ion-binding pockets. Two of them strongly bind Ca^2+^ while the other two sites coordinate Mg^2+^. These theoretical results are strongly supported by revisiting crystallographic structures that contain bpDNase I. One Ca^2+^ stabilizes the functional DNase I structure. The presence of Mg^2+^ in close vicinity to the catalytic pocket of bpDNase I reinforces the idea of a cation-assisted hydrolytic mechanism. Importantly, Poisson-Boltzmann-type electrostatic potential calculations demonstrate that the divalent cations collectively control the electrostatic fit between bpDNase I and DNA. These results improve our understanding of the essential role of cations in the biological function of bpDNase I. The high degree of conservation of the amino acids involved in the identified cation-binding sites across DNase I and DNase I-like proteins from various species suggests that our findings generally apply to all DNase I-DNA interactions.

## Author Summary

DNase I requires Ca^2+^ and Mg^2+^ for hydrolyzing double-stranded DNA. Here, we show that bovine pancreatic (bp) DNase I contains four ion-binding pockets. Two of them, previously observed in the crystallographic structure of free bpDNase I, strongly bind Ca^2+^. The other two sites bind Mg^2+^ and are described in detail for the first time. One Ca^2+^ stabilizes the functional DNase I structure. The presence of Mg^2+^ in close vicinity to the catalytic pocket of bpDNase I reinforces the idea of a cation-assisted hydrolytic mechanism. Poisson-Boltzmann-type electrostatic potential calculations demonstrate that the divalent cations collectively control the electrostatic fit between bpDNase I and DNA. Thus, this work reveals the link between cation binding and the biological function of bpDNase I. The high degree of conservation of the amino acids involved in the identified cation-binding sites across DNase I and DNase I-like proteins from various species suggests that our findings generally apply to all DNase I-DNA interactions.

## Introduction

DNase I is a ubiquitous endonuclease that cleaves the phosphodiester backbone of the DNA double helix in the presence of divalent cations, introducing single-stranded nicks through hydrolysis of the P-O3′-bond and yielding 5′-phosphorylated fragments [1], [2]. This enzyme plays a major role in digesting DNA for nutritional purposes [1], [2] but also intervenes in several other biological processes, such as DNA degradation in apoptosis [3], DNA clearance from extracellular media [4] and actin depolymerization [5]. In molecular biology, bovine pancreatic DNase I (bpDNase I), identified many years ago as a powerful footprinting agent [6] and is widely used today for *in vitro*, *in situ* and *in vivo* mapping of proteins onto genomes [7]–[10]. Finally, recombinant human DNase I has been developed clinically for treatment of pulmonary disease in patients with cystic fibrosis [11], [12]. DNase I is also under consideration for a variety of other diseases [12], including pediatric lung diseases other than cystic fibrosis [13], systemic lupus erythematosus [14], [15] and cancer [16].

DNase I, in particular bpDNase I, has been extensively studied. Its activity at physiological pH is at its highest in presence of both Ca^2+^ and Mg^2+^ [17]–[19]. DNase I activity is 100-fold lower in buffers that contain only one type of divalent cation compared to a Ca^2+^/Mg^2+^ reaction mixture [18]. Without any divalent cations, DNase I activity is almost negligible.

In addition to its effect on enzymatic activity, the importance of Ca^2+^ for the structural integrity of DNase I has long been recognized. Calcium cations protect bpDNase I from proteolytic degradation [20] and its two disulfide bridges from reduction. Equilibrium dialysis and various spectroscopic studies have led to the conclusion that bpDNase I contains two strong and several weak cation-binding sites [21], [22]. More precise information on the number and the location of divalent cations in bpDNase I can be extracted from the numerous high-resolution crystal structures available for this enzyme [23]–[25]. In particular, two calcium binding sites (site I and II) have been identified[24]. Tightly bound Ca^2+^ ions at these sites are assumed to be an integral part of functional bpDNase I. This assumption is further supported by the dramatic decrease in specific activity observed in DNase I variants devoid of Ca^2+^-binding sites I or II [26], [27].

Ca^2+^ coordination in sites I and II involves amino acids belonging to loops L1 (Leu198 to Thr211, containing site I) and L2 (Tyr97 to Pro113, containing site II), which are structured by two disulfide bridges, Cys173-Cys209 and Cys101-Cys104, respectively [24]. Ca^2+^ may further stabilize these loops and protect the disulfide bonds [28]. However, in a site I defective variant, the Cys173-Cys209 bridge, close to site I, remains resistant to β-mercaptoethanol reduction [26], demonstrating that Ca^2+^ in site I is not necessary for protecting this bridge [24]. On the other hand, Ca^2+^-containing buffers confer partial bpDNase I activity to a Cys173-Cys209 defective variant [29]. These biochemical experiments, together with conclusions deduced from the measurement of Ca^2+^ dissociation constants and Ca^2+^-induced fluorescence changes [30], cannot be interpreted without assuming the existence of more than two cation binding sites in bpDNase I. This data suggests that additional sites may potentially be essential for the hydrolytic function of DNase I.

Little has been published on the number and location of Mg^2+^-binding sites. It has been proposed that Mg^2+^ is located near the catalytic pocket and contributes to hydrolysis [31]. According to the 1DNK structure of a bpDNase I/DNA complex [32], and studies of specific chemically modified bpDNase I [33] and site-directed mutants [31], the catalytic site involves two histidines, His134 and His252 that coordinate the scissile phosphate. Similar catalytic pockets are common at the active sites in nucleases, which frequently require Mg^2+^ for their catalytic activity [34]. In addition, mutations in putative divalent metal ion-coordinating residues close to the active site of human DNase I lead to inactive variants [18]. These considerations, together with partial X-ray data [32], [35] and theoretical investigations [36] have led to the postulate that one [32], [36] or two [18], [31] divalent cations may be positioned inside or very close to the catalytic pocket of DNase I. Their functional role may be to participate in DNA hydrolysis or to stabilize the DNA phosphate groups near the cleavage site.

Thus, despite extensive biochemical and structural characterization of bpDNase I, the number and the location of cation-binding sites in free bpDNase I have still not been resolved. The location of Ca^2+^-binding sites I and II has been firmly established through both X-ray structures and biochemical studies of DNase I variants. In contrast, Mg^2+^-binding sites remain much more hypothetical. In this study, molecular dynamics (MD) simulations in explicit solvent were carried out on bpDNase I, with variation in the compositions of metal ions. We identify four cation-binding sites and demonstrate that both Ca^2+^ and Mg^2+^ are crucial for optimizing the electrostatic fit between the enzyme and the negatively charged DNA. Two Mg^2+^-binding sites located within and very close to the active site of DNase I provide the first tangible support for a cation-assisted hydrolysis process. In sum, these findings establish a direct link between cation binding and the biological function of DNase I.

## Results

### Number and location of possible cation-binding sites in bpDNase I extracted from high-resolution X-ray structures

**Figure 1:**
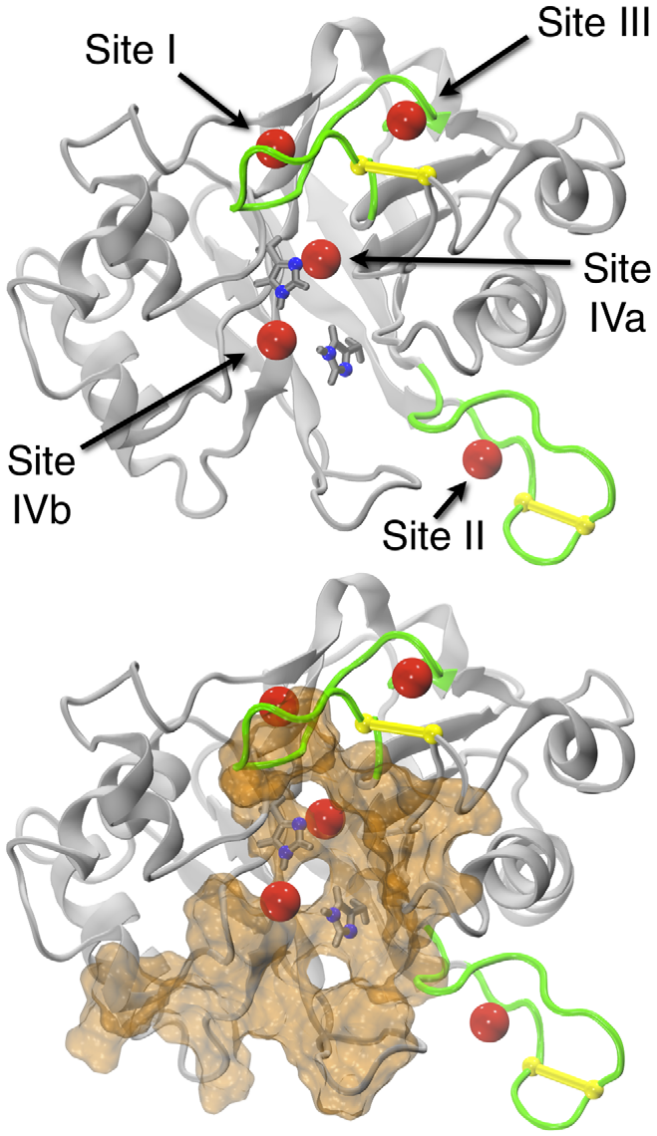
bpDNase I ion binding sites. Four sites (sites I to IVa,b) in bpDNase I are occupied by cations (red). Sites I, II and III are located in loops L1 and L2 (green), which contain disulfide bridges (yellow). Sites IVa and IVb surround the catalytic histidines (side-chains shown in gray, imidazole nitrogens in blue). Ion-binding pockets are within or near the region that interacts with DNA (orange, bottom). The highlighted DNA binding region was defined by the residues losing their solvent accessibility when complexed with DNA, as described in the Materials and Methods section.

The nine available high-resolution X-ray structures containing bpDNase I are listed in Table 1. All nine enzyme structures are very similar, with cross-root mean square deviations on Cα atoms (Cα-RMSD) lower than 0.5 Å. The structures were analyzed with respect to the number and the location of divalent cations bound to bpDNase I (Table 1). The structure of bpDNase I alone, 3DNI [24], comprises two Ca^2+^ binding sites, called sites I and II in the original publication (Figure 1). Site I was found in all of the six X-ray structures in which bpDNase I was complexed with various actin-binding motifs (1ATN, 2A40, 2A42, 2A3Z and 2A41 [25]; 2D1K [37]). Site II appeared in 3DNI and 1ATN. The frequent observation of these binding sites across free and bound bpDNase I structures confirms that Ca^2+^ tightly binds to sites I and II and should be considered as an integral part of functional bpDNase I [24]. bpDNase I in complex with actin binds a third cation, its nature depending on the buffer. This third site, called site III in Figure 1 and throughout this paper, binds Ca^2+^ when the buffer contains Ca^2+^ but not Mg^2+^ (1ATN). However, site III preferentially coordinates Mg^2+^ in presence of both Ca^2+^ and Mg^2+^ (2A40, 2A42, 2A3Z, 2A41 and 2D1K). The bpDNase I/DNA complex structures (2DNJ [35] and 1DNK [32]) were crystallized without Ca^2+^ and Mg^2+^ to limit the possibility of DNA cleavage, eluding the question of cation localization. However, crystals of bpDNase I soaking in a solution of deoxythymidine-3′,5′-diphosphate in presence of Ca^2+^ [35] and an unpublished structure of a bpDNaseI/DNA complex [32] indicate that a metal ion may be localized near the catalytic pocket.

**Table 1:** Ions bound to bpDNase I in high-resolution X-ray structures.

			DNase I cation-binding site				
PDB code	Macromolecular ligand	Divalent ions in buffer	I	II	III	IVa	IVb
3DNI	none	Ca^2+^	Ca^2+^	Ca^2+^	H_2_O	-	H_2_O
1ATN	actin	Ca^2+^	Ca^2+^	Ca^2+^	Ca^2+^	-	-
2D1K, 2A40, 2A41, 2A3Z	actin	Ca^2+^/Mg^2+^	Ca^2+^	-	Mg^2+^	-	-
2A42	actin	Ca^2+^/Mg^2+^	Ca^2+^	-	Mg^2+^	-	H_2_O
2DNJ, 1DNK	DNA	-	H_2_O	-	H_2_O	-	H_2_O

Nine high-resolution X-ray structures contain bpDNase I. The enzyme is either free or complexed with actin or DNA. The crystallization buffers contained divalent cations, except for the bpDNase I/DNA complexes. The bpDNase I binding sites (Figure 1) can be occupied by divalent cations or water molecules.

All of these observations are supported by the analysis of bound crystallographic water molecules (Table 1). Accordingly, bound water molecules are observed at sites I (2DNJ and 1DNK) and III (3DNI, 2DNJ and 1DNK). Interestingly, they are also present near the catalytic pockets in 3DNI, 2DNJ and 1DNK (Figure 1), areas which have previously been proposed as a possible fourth cation-binding site [32], [38]. This hypothetical ion-binding site is called site IV herein.

Overall, these analyses show that at least three sites potentially bind divalent cations in bpDNase I. In addition to the well-known sites I and II, with high affinities for Ca^2+^, the detection of site III is particularly interesting since it seems to preferentially bind Mg^2+^. It remains to be verified whether this site can stabilize Mg^2+^ in actin-free bpDNase I. The existence of a fourth putative site located near the bpDNase I catalytic pocket, possibly occupied by Mg^2+^, is only supported by the presence of bound water molecules in three structures and thus must be explored *de novo*.

### Structural drift in bpDNase I simulations

The confirmed or putative cation-binding sites identified above were further investigated using molecular dynamics simulations of bpDNase I carried out in the presence of various types of metal ions in explicit solvent. In all seven simulations, the starting positions of Na^+^ around the enzyme were determined using a Coulombic potential grid and, importantly, were not initially located in cation-binding sites. The characteristics of the seven simulations are summarized in Table 2. The Cα-RMSD values calculated between the free 3DNI bpDNase I and the simulated structures ranged from 1.0 (Sim3) to 1.7 (Sim 1) ±0.1 Å (Table 2), indicating that no major structural reorganizations occurred throughout the trajectories. The low standard deviations (0.1 Å, Table 2) attest to the stability of these simulations. In general, all simulated structures remained reasonably close to the 3DNI crystal structure.

**Table 2:** MD simulations performed on bpDNase I.

	Bound ions					
Name	Site I	Site II	Site III	Site IVa	Site IVb	Cα-RMSD^av^
Sim1	Na^+^	Na^+^	Na^+^	-	Na^+^	1.7 (0.1)
Sim2	Ca^2+^	Ca^2+^	Na^+^	Na^+^	-	1.4 (0.1)
Sim3	Ca^2+^	Ca^2+^	Mg^2+^	Na^+^	-	1.0 (0.1)
Sim4	Ca^2+^	Ca^2+^	Na^+^	Mg^2+^	-	1.2 (0.1)
Sim5	Ca^2+^	Ca^2+^	Mg^2+^	Mg^2+^	-	1.5 (0.1)
Sim6	Ca^2+^	Ca^2+^	Mg^2+^	-	Mg^2+^	1.5 (0.1)
Sim7	Ca^2+^	Ca^2+^	Mg^2+^	Mg^2+^	Mg^2+^	1.5 (0.1)

The bpDNase I simulations were performed with solvent containing Na^+^, Na^+^ and Ca^2+^, or Na^+^, Ca^2+^ and Mg^2+^. The ions bound at sites I, II, III and IVa,b (Figure 1) are given for each simulation. The Cα-RMSD^av^ values (Å) are calculated between the snapshots extracted from the last 20 ns of each trajectory and the 3DNI X-ray structure. Standard deviations of Cα-RMSD^av^ are given in parentheses.

### Number, location and coordination of cations in bpDNase I simulations

**Figure 2:**
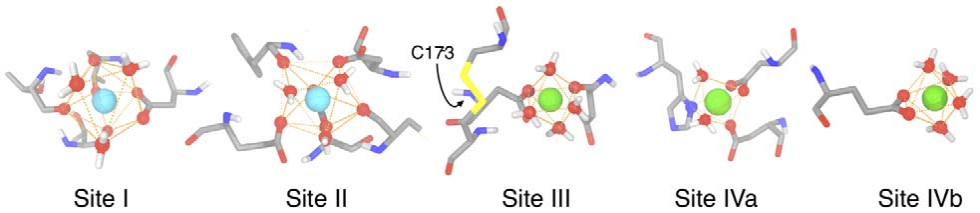
Coordination spheres of Ca^2+^ and Mg^2+^ bound to bpDNase I. Ca^2+^ (cyan) at sites I and II are involved in coordination spheres with tricapped trigonal prismatic geometry. The coordination spheres of Mg^2+^ (green) at sites III, IVa and IVb are octahedral. Cys173 interacts with a water molecule belonging to the coordination sphere of site III. The amino acids involved in the coordination spheres are listed in Table 2.

Sites I and II in bpDNase I, identical to their X-ray counter-parts, were very strong cation-binding pockets, always occupied by ions throughout the trajectories (Table 3). Na^+^ spontaneously occupied these sites in the absence of Ca^2+^ (Sim1). Ion coordination was optimal for both Na^+^ and Ca^2+^ (Table 4). Sites I and II were further strengthened by binding Ca^2+^ (Figure 2), given its maximal coordination number (CN_max_) of 9 [39], higher than for Na^+^ (CN_max_ of 6 [39]). In addition, compared to Na^+^, the participation of Cys101 in the Cys101-Cys104 bridge reinforced the amino acid side-chain coordination of Ca^2+^ at site II (Tables 3 and 4). Both the amino-acid constitution and the tricapped trigonal prismatic geometry of sites I and II are identical to those of crystallographic structures.

**Table 3:** bpDNase I binding site occupancy by cation species.

Site	AA_cs_	%t_occ_ (Na^+^)	%t_occ_ (Ca^2+^)	%t_occ_ (Mg^2+^)
Site I	Asp201	92	99	N.A.
	Thr203	99	99	N.A.
	Thr205	100	100	N.A.
	Thr207	100	100	N.A.
Site II	Asp99	95	100	N.A.
	Cys101	0	100	N.A.
	Asp107	66	97	N.A.
	Phe109	97	100	N.A.
	Glu112	95	72	N.A.
Site III	Asp172	68	N.A.	100
	Asp198	72	N.A.	100
Site IVa	Asp168	78	N.A.	100
	Asp212	99	N.A.	100
	His252	96	N.A.	100
Site IVb	Asn7	95	N.A.	25
	Ile8	96	N.A.	0
	Glu39	55	N.A.	100

**Figure 3:**
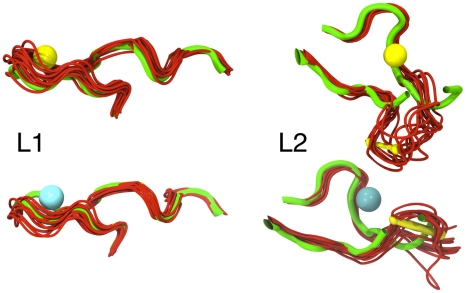
L1 and L2 dynamics. Ten snapshots (red) of L1 (left) and L2 (right) loop structures from Sim1 or Sim7 were superimposed on their counterparts in the 3DNI X-ray structure (green). Site I in L1 and site II in L2 bind either Na^+^ (dark blue, top panels) or Ca^2+^ (cyan, bottom panels). L2 contains one disulfide bridge (yellow).

Amino acids involved in the coordination sphere (AA_cs_) of the four bpDNase I cation-binding sites (Figure 1 and 3). The percentage of simulation time (%t_occ_) corresponding to effective coordination of each cation was calculated for the last 20 ns of the relevant trajectories (Table 2). This amounts to 20 ns for Na^+^ (I, II, IVb), 40 ns for Na^+^ (IVa) and Mg^2+^ (IVb), 60 ns for Na^+^ (III) and Mg^2+^ (IVa), 80 ns Mg^2+^ (III) and 120 ns for Ca^2+^. The interaction between Mg^2+^ and His252 is either direct or mediated by a water molecule. N.A.: not applicable.

**Table 4:** Cation coordination number in bpDNase I binding sites.

Site	CN	Na^+^	Ca^2+^	Mg^2+^
Site I	aa	5.1	5.4	N.A.
	water	1.2	3.6	N.A.
	total	6.3	9.0	N.A.
Site II	aa	3.9	6.6	N.A.
	water	2.0	2.4	N.A.
	total	5.9	9.0	N.A.
Site III	aa	2.3	N.A.	2.0
	water	3.5	N.A.	4.0
	total	5.8	N.A.	6.0
Site IVa	aa	4.3	N.A.	4.3
	water	1.6	N.A.	1.7
	total	5.9	N.A.	6.0
Site IVb	aa	3.0	N.A.	2.0
	water	3.0	N.A.	4.0
	total	6.0	N.A.	6.0

The coordination number (CN) of the cations bound to the four bpDNase I sites (Figure 1 and 3) were calculated for the relevant trajectories (Tables 2 & 3). The total coordination number (total) was broken down into contributions from amino acids (aa) and water molecules (water). N.A.: not applicable.

Site III, close to site I (Figure 1), favored Mg^2+^ in the X-ray structures of bpDNase I in complex with actin (Table 1). Our simulations show that Na^+^ could spontaneously bind to site III in actin-free bpDNase I (Tables 2 and 3, Sim1, Sim2 and Sim4). Replacing Na^+^ with Mg^2+^ in Sim3 and Sim5–7 demonstrated that this site could also bind Mg^2+^ with an octahedral geometry, which has also been observed in the relevant crystallographic structures [25] (Tables 3 and 4, Figure 2). The coordination numbers were maximal for both Na^+^ and Mg^2+^ (CN_max_ of 6 [40]). Nevertheless, the maximal occupation time (100%) observed for Mg^2+^ indicates that this divalent cation was strongly bound to site III. Independently of the nature of the ion, only two bpDNase I residues were involved in the coordination sphere (Table 3, Figure 2), which was completed with water molecules. This feature, in line with crystal structures of bpDNase I/actin complexes [25], may imply that site III is weaker than sites I and II.

The existence of a fourth site (site IV) specific to Mg^2+^ has been suspected [31], [32], [38] but never confirmed. A recent computational approach dedicated to the prediction of potential Ca^2+^ binding sites detected one such pocket near the DNase I active site [36]. In the present study, site IV was discovered through the spontaneous binding of Na^+^ in Sim1 and Sim2. Site IV is a double-binding site, with two sub-sites, called IVa and IVb. The coordination sphere of site IVa involves the catalytic His252 residue. The ion at site IVb is very close (∼5 Å on average) to the second catalytic residue, His134, which is implicated in cleavage activity. Sim3–7 show that both sub-sites were able to retain Mg^2+^ (Tables 3 and 4, Figure 2). According to its protein coordination (Table 4), Site IVa appeared stronger than sites III or IVb. More refined computational models would however be required to assess the relative strength of these binding sites with confidence. At first, sub-sites IVa and IVb appeared to be mutually exclusive, since they were never both spontaneously occupied simultaneously in Sim1-6. This was tested with trajectory Sim7 by constructing a starting point with two Mg^2+^ ions present at sites IVa and IVb. This simulation demonstrates that these sites could be simultaneously occupied by two divalent cations, keeping their intrinsic coordination characteristics.

In summary, four cation-binding sites were identified in free bpDNase I. The strong sites I, II and IVa, as well as the two additional sites III and IVb with fewer coordinating protein side-chains, were able to bind divalent cations.

### Validation of the theoretical cation binding sites

The prediction of cation binding sites by molecular dynamics can potentially be biased by incomplete sampling of ion positions. In MD simulations with Ca^2+^ and Mg^2+^, these divalent cations were directly located at the sites where Na^+^ spontaneously binds. Conversely, Na^+^ ions were randomly distributed around DNase I, yet artifacts may have been caused by attraction from the closest negatively charged side-chains. In order to make sure that no such artifacts apply in the present case, we used two complementary approaches to systematically explore all possible cation pockets on the entire surface of DNase I. First, ion locations were interactively investigated with the “MyPal” approach that allows steering ions within DNase I electrostatic potential maps using a haptic device [41]. Second, the CHED server for predicting metal binding sites in proteins [42] was used to systematically scan all DNase I crystal structures for cation pockets (see Table S1 in Supplemental Data). In both cases, the cation binding sites corresponded exactly to those highlighted by the MD simulations and, importantly, no additional pockets could be detected, further validating our findings.

The very high residence times of Mg^2+^ in MD simulations could be another issue. Indeed, this ion, due to its +2 charge and small radius, could be artificially trapped by DNase I. However, the fact that Na^+^ binds to sites III and IVa,b over the whole Sim 1–3 trajectories precludes the possibility of a specific bias towards Mg^2+^ in the present case.

The characteristics of theoretical sites I, II and III perfectly parallel those observed in the crystallographic structures containing DNase I, as mentioned in the previous section. Their existence is thus well attested. In order to obtain equally sound experimental evidence for the existence of site IV, we revisited the X-ray experimental electron density maps of the highest resolution structures 2A40 and 2A42. In both maps, significant densities are either unattributed (2A40) or attributed to a water molecule (2A42) at the location of site IVb. These perfectly match a divalent cation interacting with Glu39 and one (2A40) or five (2A42) water molecules (for details, Figure S1 and the comments in the Supplemental Data). These observations support earlier crystallographic considerations [32], [35] suggesting that a metal ion might be bound near the catalytic site, in particular to Glu39. The existence of site IVb can thus be attested from the original X-ray data. No similar feature is found in the region of site IVa. However, several site-directed mutagenesis experiments on residues surrounding His134 and His252 demonstrated that single mutations of Glu39, Asp168 or Asp212 resulted in very low activities on DNA, for either bovine [31] or human [18] DNase I. The effects of these mutations add strength to the case for the existence of sub-site IVb (Glu39), and also indirectly add evidence for a site IVa, involving Asp168 and Asp212.

### Structural effects of ions at sites I, II and III

Sites I and II are located in loops L1 (Leu195 to Tyr211) and L2 (Tyr97 to Pro113) respectively (Figure 1). The structural stability of L1, reflected by the low temperature factors in 3DNI [24] and the low root mean square fluctuation (RMSF) values (0.7±0.1 Å on average) in our simulations, was not affected by the nature of the bound ion (Figure 3). Conversely, the presence of ions affected L2, which was more flexible than L1 (Figure 3). Na^+^ in site II led to higher L2-RMSF values (1.3±0.1 Å on average) than Ca^2+^ (0.9±0.1 Å on average). The simulations further highlight that when Na^+^ was bound at site II, the Gly100-Cys104 region significantly deviated from the configuration observed with Ca^2+^ bound at this site (Figure 3). This suggests that Ca^2+^ at site II restricts L2 flexibility and stabilizes one of at least two possible conformations of L2.

Mg^2+^ at site III was coordinated to Asp172, protecting the Cys173-Cys209 bridge from reduction. Accordingly, one water molecule involved in the Mg^2+^ coordination sphere was also interacting with Cys173 (Figure 2). This water molecule, firmly bound by both Mg^2+^ and Cys173, did not exchange with the solvent and partially shielded the Cys173 sulphur atom, reducing its accessibility. This situation was specific to Mg^2+^, since the coordination sphere of Na^+^ at site III was formed by exchangeable water molecules that could not protect Cys173. Cation binding at the previously unknown site III provides a comprehensive interpretation of the resistance of the Cys173-Cys209 disulfide bridge to β-mercaptoethanol attack in a site I-defective bpDNase I variant [26].

Individually, cations stabilized the enzyme's structure, either directly (Ca^2+^ at site II) or indirectly (Mg^2+^ at sites III and IV).

### Possible involvement of ions at sub-sites IVa and IVb in the catalytic mechanism

**Figure 4:**
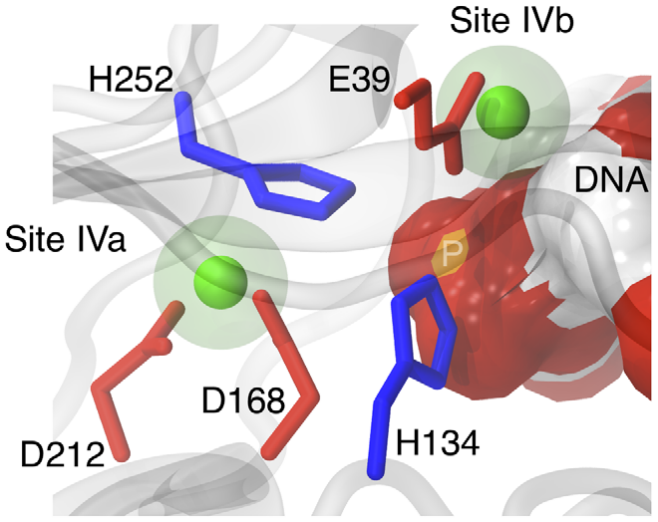
Detailed view of Site IV. A close-up view of the double magnesium binding site IVa and IVb, where DNA from the 1DNK complex was modeled in by superimposition of the protein chains from our simulation and from the crystal structure. Acidic side-chains are shown in red, histidines in blue, magnesium in green. The size of the cations is indicated by a transparent van der Waals sphere to assess their possible contact with DNA. The DNA surface is represented colored by underlying atom type, red for oxygen, orange for phosphorus, blue for nitrogen, white for carbon.

The proximity between the catalytic site and the double-binding site IV, involving two aspartates, one catalytic histidine and one glutamate, suggests that Mg^2+^ at sub-sites IVa and IVb may assist in the hydrolysis of the DNA phosphodiester bond. The approximate location of DNA with respect to Site IV is depicted in Figure 4. This type of catalytic mechanism involving two metal ions is now assumed for numerous hydrolases [43]–[45]. For instance, catalysis in type II restriction endonucleases may involve two Mg^2+^ ions coordinated by aspartate and glutamate [43], [46]. In DNase I, the possible double occupancy of sub-sites IVa and IVb by two Mg^2+^ ions would specifically mean that the DNase I hydrolytic mechanism involves two metal ions, as previously proposed [31]. In our simulations, Mg^2+^ at sub-site IVa was particularly buried (accessibility of the ion devoid of coordinating water molecules of 2.3±0.6 Å^2^) compared to cations at sites I, II and III (accessibility of 13.7±3.3 Å^2^, on average). This suggests that in presence of DNA, Mg^2+^ is unlikely to directly bind to the scissile DNA phosphate group. However, this ion coordinates His252 either directly or indirectly *via* a non-exchangeable water molecule and may thus stabilize this catalytic residue in a position that favors hydrolysis. In contrast, Mg^2+^ at sub-site IVb was bound close to His134 without involving any direct contact but was largely exposed (accessibility of 21.0±1.2 Å^2^). This particularity may allow Mg^2+^ at sub-site IVb to interact with DNA to correctly position the non-esterified oxygen of the phosphate group, as previously postulated [31].

At the present stage, our study cannot reveal further details of the bpDNase I hydrolysis process. However, the discovery of site IV strengthens the hypothesis that Mg^2+^ ions are directly implicated in the enzymatic function of DNase I.

### Collective effects of ions on the electrostatics of the bpDNase I/DNA interface

In addition to these diverse roles, we suspected that ions may interfere with the bpDNase I/DNA interaction. Any favorable interaction between two charged macromolecules requires an electrostatic fit between them. Nucleic acid molecules are negatively charged, mainly owing to their phosphate groups. Their interaction with proteins thus requires positively charged protein surfaces [47]–[49]. Regarding the DNase I enzyme, hyperactive variants of the human protein have been obtained by introducing additional positively charged amino acids at the DNA binding interface [50]. An investigation of the effect of ions on the electrostatic properties of the bpDNase I surface is all the more relevant since the bpDNase I ion binding pockets are either in (site IV) or very close to (sites I, II and III) the region at the interface with the DNA substrate (Figure 1).

Poisson-Boltzmann electrostatic potential maps were calculated for bpDNase I structures extracted from simulations Sim3–7, focusing on the region where DNA interacts with the enzyme. According to the 3DNI, 2DNJ and 1DNK structures, this region involves 24 amino acids (listed in Materials and Methods). In addition to the enzyme without any bound ions, we also considered bpDNase I with various combinations of bound ions, with sites I and II always coordinating Ca2+.

**Figure 5:**
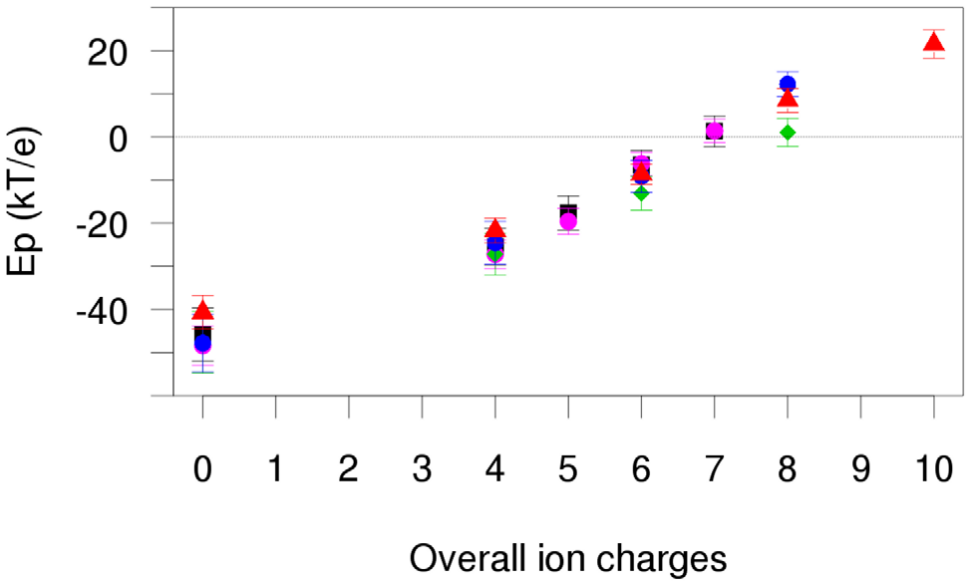
Effect of bound ions on the electrostatic potential of the bpDNase I region that interacts with DNA. Electrostatic potentials (Ep, in kT/e units) of the bpDNase I region involved in the interaction with DNA were calculated on ten snapshots extracted from Sim3 (black), Sim4 (pink), Sim5 (green), Sim6 (blue) and Sim7 (red). Ep values are ordered as a function of the number of charges carried by the ions at sites I, II, III or IVa,b. Error bars represent standard deviations.

**Figure 6:**
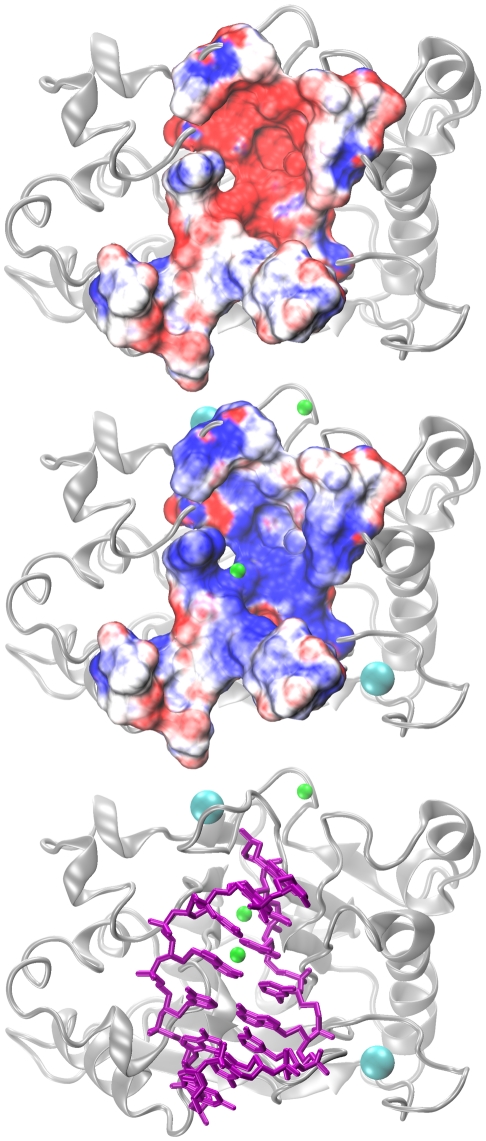
Effect of bound ions on the electrostatic potential surface of the bpDNase I region that interacts with DNA. Electrostatic potential maps colored from -5 kT/e in red to +5 kT/e in blue for the bpDNase I region involved in the interaction with DNA. The top panel shows the map obtained in the absence of any bound cations, the central panel shows the map for sites I & II occupied by Ca^2+^ (cyan), and III, IVa and IVb by Mg^2+^ (green). The bottom panel indicates the location of DNA (magenta) at its binding site on DNase I with respect to the ion binding sites.

The electrostatic potential of the interface was extremely sensitive to the number of charges contributed by the ions (Figures 5 and 6). Despite a moderate net charge of -1 (against -9 for the whole protein), the bpDNase I interface displayed a very negative electrostatic potential in the absence of counter-ions, thus inhibiting DNA binding. Adding ions to the identified binding sites progressively lowered the negative surface charge. The contribution of the deeply buried Mg^2+^ at sub-site IVa was lower than for the other bound ions (Figure 5, Sim5). The presence of eight positive charges was required to reverse the enzyme's surface charge to positive values. Since both sites I and II are occupied by Ca^2+^, at least two additional divalent cations are required to enable DNA binding.

The existence of crystallographic DNA/DNase I complexes (2DNJ [35] and 1DNK [32]) seems in contradiction with this result. Indeed, these two complexes were crystallized in a medium devoid of divalent cations to try to prevent DNA hydrolysis. Yet, the presence of monovalent ions, very difficult to detect by X-ray crystallography, cannot be ruled out. Such monovalent cations could limit the electrostatic repulsion between the two partners and may also explain why the DNA is cleaved in the 2DNJ structure. In addition, water molecules may contribute to electrostatic screening.

Poisson-Boltzmann calculations show that ions bound to DNase I can collectively influence the electrostatic potential of the bpDNase I region involved in the DNA interface, and thus drive the electrostatic fit between the enzyme and the DNA substrate.

### Conservation of the residues involved in bpDNase I ion binding sites

The importance of individual amino acids in bpDNase I cation-binding sites can be further assessed by comparing the sequences from DNase I and DNase I-like proteins of various species (from 30 for DNase I to 11 for DNase I-like 2). DNase I-like proteins include three variants. The sequences and their percentages of identity with bpDNase are listed in Table S2, Table S3, Table S4, Table S5 (in Supplemental Data). Using bpDNase I as a reference, the DNase I sequence identities ranged from 43 to 93%, except for one sequence that exhibited a very low identity of 20%. DNase I-like proteins shared from 22 to 58% sequence identity with bpDNase I.

**Figure 7:**
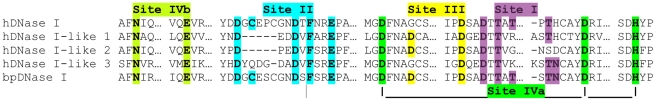
Conservation of cation binding sites in bpDNase I, human DNase I and DNase I-like proteins. Sequences of the human DNase I and DNase I-like 1, 2 and 3 proteins were aligned with the bovine pancreatic DNase I sequence. The alignment highlights the bpDNase I cation-binding sites I, II, III, IVa and IVb. Most of the corresponding amino acids belonging to human enzymes are either identical or similar to those of bpDNase I.

The conservation of the bpDNase I cation-binding sites is shown in Table 5 and illustrated for the human DNase I in Figure 7. Sites II, IVa and IVb are almost perfectly conserved, apart from Cys101 in site II. Asp201 and Thr203 in site I are also well conserved. The three other site I residues show more substitutions, but conserve their polarity. For instance, the bpDNase I motif Thr207-Asn208 is often inverted to Asn207-Thr208. Site III conservation is less straightforward. The Asp198 residue is common to the different DNase I proteins and to various species, but Asp172 is replaced by Gly in DNase I and DNase I-like 3.

**Table 5:** Conservation of the residues involved in bpDNase I ion binding sites across four DNase I families in various species.

		DNase I		DNase I-like 1		DNase I-like 2		DNase I-like 3	
Site	Residue	I (%)	S (%)	I (%)	S (%)	I (%)	S (%)	I (%)	S (%)
I	Asp201	97		94		91		94	
	Thr203	93		100		91		82	
	Thr205	47	Ser: 40	0	Ala: 88	0	Gly: 100	0	polar/charged: 88
	Thr207	77	polar: 20	94		9	Ser: 82	59	Asn: 4
	Asn208	17	polar: 80	19	His: 75	0	polar/charged: 91	12	polar/charged: 68
II	Asp99	97		94		100		82	
	Cys101	73		0	gap: 100	0	gap: 100	0	polar: 80
	Asp107	97		94		100		100	
	Phe109	97		100		100		88	
	Glu112	90		100		100		88	
III	Asp172	30	Gly: 63	94		91		47	Gly: 41
	Asp198	97		100		91		100	
IVa	Asp168	97		100		91		100	
	Asp212	100		100		91		100	
	His252	97		94		100		100	
IVb	Asn7	97		100		82		100	
	Glu39	97		100		91		100	

The bpDNase I sequence was aligned with 30 non-redundant DNase I, 16 DNase I-like 1, 11 DNase I-like 2 and 17 DNase I-like 3 sequences from different species. I (%) corresponds to the percentage of identity for each of the amino acids involved in each of the four bpDNase I cation binding sites, I, II, III and IVa,b. For relevant cases, S (%) is the percentage of the amino acid or the type of amino acid substituting those of bpDNase I.

Overall, the cation-binding sites in bpDNase I are as well conserved as the residues involved in DNase I/DNA contacts [18], [51], especially sites I, II and IVa and b. This strongly supports the critical role of these sites for the biological function of DNase I.

## Discussion

The aim of this work was to investigate the ability of bpDNase I to bind divalent cations, Ca^2+^ and Mg^2+^, and to elucidate their possible roles in bpDNase I function.

In several simulations, we used monovalent sodium ions as a reference state, as they may occupy any of the potential binding sites. It should be noted that NaCl effectively inhibits DNase I activity [17], [52], which can be reversed by addition of divalent ions [53]. This effect is related to the ionic strength of the system as well as to the positive charges present on the protein [54], which is fully compatible with the trends observed in our electrostatic surface calculations. The experimental findings underline the importance of electrostatics and show a particularly interesting mechanistic effect of engineering positively charged residues into the enzyme.

Molecular dynamics simulations reveal that four sites are able to bind Ca^2+^ (sites I and II) or Mg^2+^ (sites III and IVa and b). The existence of sites I, II and III is demonstrated by the X-ray structures of free (sites I and II) and actin-bound bpDNase I (sites I, II and III). Reexamination of the 2A40 and 2A42 electron density maps validates sub-site IVb. Sub-site IVa is indirectly but firmly confirmed by site-directed mutations [31]. The occupation time, the coordination characteristics, and the conservation of amino-acids involved in these sites show that sites I, II and IV correspond to strong bpDNase I cation-binding pockets, while site III is weaker.

At site II, Ca^2+^ acts on the folding of the L2 loop and reduces its mobility. At site III, Mg^2+^ is located very close to the Cys173-Cys209 bridge and protects this essential structural element from reduction. Site IV corresponds to two sub-sites, IVa and IVb, with an ion either coordinating (sub-site IVa) or close (sub-site IVb) to the two histidine residues involved in the DNA cleavage process. The discovery of site IV is a first step towards a comprehensive understanding of the bpDNase I enzymatic mechanism. In addition to these local functions, bound ions collectively modify the electrostatic potential of the bpDNase I region implicated in DNA binding. By introducing positive charges, they compensate for the intrinsic repulsion between DNase I and DNA, both negatively charged. A similar effect may have been achieved by engineering Human DNase I mutants, introducing additional positive charges at the DNA binding domain [54].

Beyond the current investigation of Mg^2+^ and Ca^2+^ binding, this study also opens the prospect to address the effects of other divalent metal ions, for instance Mn^2+^, able to enhance DNase I activity [17], [53]. However, more refined computational methods may be required for such investigations in order to properly account for the differences between such cations.

Our results are consistent with a recent study highlighting how the prediction of ion binding sites may improve our understanding of structure-based protein functions [36]. The characterization of the bpDNase I cation-binding sites reveals how these bound ions can contribute to bpDNase I function at various levels. The remarkable conservation of these binding sites across diverse proteins belonging to the DNase I family and across different species suggests that our findings are significant for DNase I proteins in general. Building better models of DNase I structure and action are essential, given the role of DNase I in numerous fundamental biological processes and its wide-spread use in biochemical and medical contexts.

## Materials and Methods

### Molecular dynamics simulations

A summary of all MD simulations is given in Table 2. Simulations were performed using the AMBER 8 program [55] with the Parm99 force field (ff99 [56]). DNase I was neutralized with various cations (Na^+^; Na^+^ and Ca^2+^; Na^+^, Ca^2+^ and Mg^2+^) and hydrated with TIP3P water molecules using a truncated octahedron as simulation box. The box size is 15 Å in all directions. Simulations were performed at constant temperature (300 K) and pressure (1 bar) using a Berendsen coupling algorithm [57]. The integration time–step was 2 fs and covalent bonds involving hydrogen were constrained using SHAKE [58]. Long-range electrostatic interactions were treated using the particle mesh Ewald (PME) approach [59] with a 9 Å direct space cut-off. The non-bonded pair list was updated every 25 steps and the center of mass motion removed every 10 ps.

Water molecules and cations were energy-minimized and equilibrated in the NVT ensemble at 100 K for 100 ps, with the protein constrained. The entire system (bpDNase I, water molecules and ions) was then heated from 100 to 300 K in 10 ps by 5 K increments with harmonic restraints of 5.0 kcal mol^−1^ Å^−2^ on the solute atoms. The simulations were continued in the NPT ensemble, without a noticeable change in volume. Subsequently, production runs lasting 25 ns were carried out.

### Starting points for the simulations

The same initial configuration was used for Sim1 and Sim2. This starting point was constructed from the crystal structure of the bpDNase I enzyme at 2 Å resolution (PDB code 3DNI [24]). In 3DNI, the atomic positions of Glu99 and Ser100 belonging to the flexible L2 loop could not be resolved. Using AMBER, we built and relaxed a complete protein including these two residues. Sim3–7 starting structures were derived from the structure of Sim2 at 20 ns.

A Coulombic potential grid was used to determine the initial positions of Na^+^ ions. Ca^2+^ locations in Sim3–7 were those observed in 3DNI. In addition to these two 3DNI Ca^2+^ sites, two strong Na^+^ binding sites were observed in Sim1 and Sim2. These bound Na^+^ ions were replaced by Mg^2+^ in Sim3–7.

In all simulations, the two histidines involved in the catalytic pocket, His134 and His252, were in their neutral form to test a potential cation-binding site under favorable conditions. This was consistent with calculations carried out on 3DNI devoid of cations with the WhatIf program [60] that yielded pKa estimates of ∼7.3 for both His134 and His252.

### Radial distribution functions (RDF)

All radial distribution functions were computed using the g_rdf analysis module of the Gromacs software suite. RDF analysis was used to determine ion–oxygen distributions and coordination numbers for Na^+^, Ca^2+^ and Mg^2+^. These coordination numbers were calculated for all ions bound to bpDNase I in each trajectory. Fixed distances of 3.2 Å for Na^+^ and Ca^2+^ and 2.9 Å for Mg^2+^ were used to define the outer limit of the first solvation shell [40], [61].

### Electrostatic potential calculations

Electrostatic potential maps were calculated with the Adaptive Poisson-Boltzmann Solver (APBS) [62] on 10 snapshots extracted from the last 10 ns of the trajectories, using APBS default parameters (physiological salt concentration of 150 mM, temperature of 298.15 K, solvent dielectric of 78.4, and solute dielectric of 2). Van der Waals radii and partial charges of both the protein and the Ca^2+^, Na^+^ and Mg^2+^ ions were those of the AMBER ff99 force field. Solute charges were distributed onto grid points using a cubic B-spline discretization. The molecular surface was defined by the interface between a 1.4 Å solvent probe, corresponding to the radius of a water molecule, and the solute van der Waals radii.

These calculations focused on the bpDNase I region corresponding to the bpDNase I/DNA interface. The amino acids belonging to this interface were determined by comparing the amino acid accessibilities in free (3DNI [24]) and bound DNase I (2DNJ [35] and 1DNK [32]) with Naccess [63]. The interface includes a total of 24 residues, Arg9, Thr10, Gly12, Glu13, Thr14, Glu39, Arg41, Asp42, Ser43, Asn74, Ser75, Tyr76, Arg111, Ala136, Pro137, Asp168, Asn170, Tyr175, Thr203, Thr205, Thr207, Tyr211 and the two catalytic histidines, His134 and His252.

Various combinations of ions were tested, from naked (no ion) to maximally charged DNase I, *i.e.* with a total of ten positive charges. Naked or partially charged DNase I structures were obtained by removing ions from the snapshots. In the APBS calculations including ions, sites I and II always coordinated Ca2+. Despite the well known difficulty of obtaining accurate potentials with explicit ions [64], these calculations were consistent across the trajectories (see Figure 5 in the “Results” section). We conclude that these results provide a good estimate of the effect of ions on the DNase I electrostatic potential.

### Alignments

The first 500 sequences homologous to bpDNase I were extracted from the Basic Local Alignment Search Tool (BLAST) [65]. They were then sorted into four families, DNase I, DNase I-like 1, 2 and 3, each set containing non-redundant sequences. The alignment with the bpDNase I sequence was carried out using ClustalW2 [66].

### Analysis and graphics

Secondary structure elements were identified using the STRIDE method by Heinig and Frishman [67]. Accessibilities were calculated using Naccess [63] and a radius probe of 1.4 Å. Graphical representations were prepared with VMD [68]. Standard conformational analysis was carried out using tools from the GROMACS package, the Ptraj module of AMBER and the PTools library [69]. Statistical and data analyses were performed using the R statistical software package [70].

## Supporting Information

Figure S1 Reinterpretation of the 2A40 and 2A42 crystal structures. Figure S1 is related to Table 1. Identified divalent metal ion binding sites in the 2A40 (a) and 2A42 (b) structures. In both cases the ion (green) is coordinated by Glu39 and 1 to 5 water molecules (red). Densities are contoured at 4σ (black). In (b), we also contoured at 3σ (blue) as this threshold was used to assign the water molecule at 2.5 Å distance.
(0.97 MB DOC)

Table S1 Ion binding sites predicted via the CHED server. This Table is related to Figure 1. The CHED server, available on line (http://ligin.weizmann.ac.il/~lpgerzon/mbs4), refers to Babor, M., Gerzon, S., Raveh, B., Sobolev, V. and Edelman, M. (2008) Prediction of transition metal-binding sites from apo protein structures. Proteins, 70, 208-217. N.O. stands for not observed. X marks the binding sites that were identified. ^a^ Both subsites IVa and IVb are identified as a single binding site.
(0.04 MB DOC)

Table S2 Thirty non-redundant DNase I sequences from various species. This Table is related to Table 5. The sequences homologous to bpDNase I correspond to precursors or mature proteins attributed to the DNase I family. The protein lengths are those of the original selected sequences.
(0.05 MB DOC)

Table S3 Sixteen non-redundant DNase I-like 1 sequences from various species. This Table is related to Table 5. The sequences homologous to bpDNase I correspond to precursors or mature proteins attributed to the DNase I-like I family. The protein lengths are those of the original selected sequences.
(0.04 MB DOC)

Table S4 Eleven non-redundant DNase I-like 2 sequences from various species. This Table is related to Table 5. The sequences homologous to bpDNase I correspond to precursors or mature proteins attributed to the DNase I-like 2 family. The protein lengths are those of the original selected sequences.
(0.03 MB DOC)

Table S5 Seventeen non-redundant DNase I-like 3 sequences from various species. This Table is related to Table 5. The sequences homologous to bpDNase I correspond to precursors or mature proteins attributed to the DNase I-like 3 family. The protein lengths are those of the original selected sequences.
(0.04 MB DOC)
