# Supplementary material for: How Cations Can Assist DNase I in DNA Binding and Hydrolysis
Source: PLoS Comput Biol. 2010 Nov 18;6(11):e1001000. doi: 10.1371/journal.pcbi.1001000 (PMC2987838; doi:10.1371/journal.pcbi.1001000)
Supplement: Figure S1 — Reinterpretation of the 2A40 and 2A42 crystal structures. Figure S1 is related to Table 1. Identified divalent metal ion binding sites in the 2A40 (a) and 2A42 (b) structures. In both cases the ion (green) is coordinated by Glu39 and 1 to 5 water molecules (red). Densities are contoured at 4σ (black). In (b), we also contoured at 3σ (blue) as this threshold was used to assign the water molecule at 2.5 Å distance. (0.97 MB DOC) [file pcbi.1001000.s001.doc]

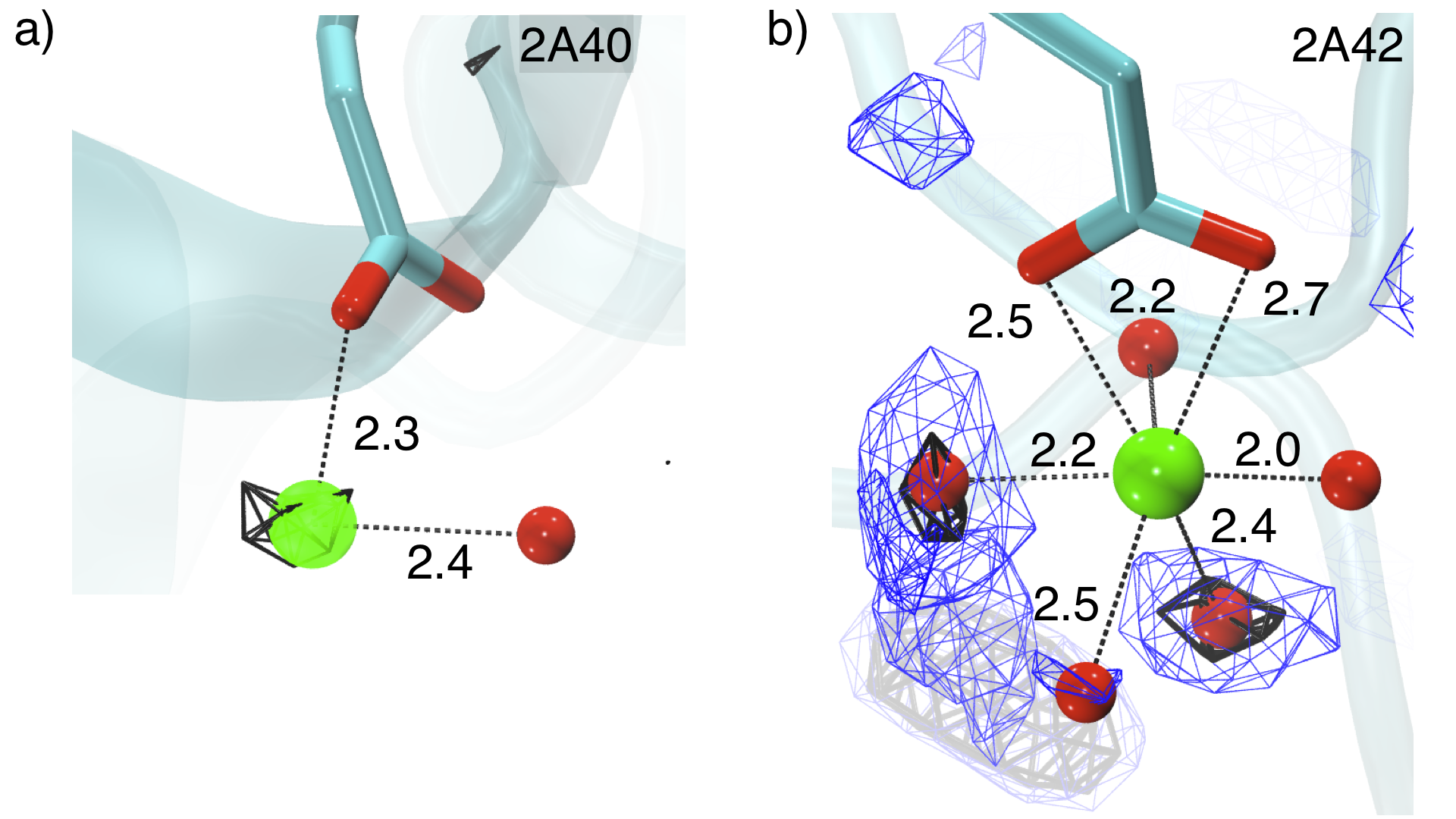


**Commentary**:

The 2A40 and 2A42 structures and maps were retrieved from the EDS server at Uppsala (1). Site IV was re-examined for possible divalent metal cations, either at unattributed density peaks or at the location of previously attributed crystallographic water molecules. In the 2A40 structure, a previously unattributed residual density of 4σ was assigned to a divalent metal cation, coordinated by Glu39 and the 1348 water molecule (Figure S1a). In the 2A42 structure, the 1391B water molecule was re-interpreted as a divalent metal cation, given its very short distances to two other water molecules, 1401B and 1310B. Three residual density peaks were attributed to coordinating water molecules completing the coordination sphere together with Glu39 (Figure S1b).

Exploratory re-refinement of the 2A42 model indicates its compatibility with the original electron density map. It is difficult to unambiguously assign the nature of the metal cation, which at this resolution could be either a magnesium or a calcium. However, coordination by a single protein side-chain complemented by several water molecules rather indicates the presence of a magnesium ion as documented by statistical analysis of metal ion binding sites in the PDB (2).

Further ample discussion of magnesium and calcium ion coordination in crystallographic structures and of intrinsic properties of their coordination sphere can be found in the literature (2-4).

1. Kleywegt, G.J., Harris, M.R., Zou, J.Y., Taylor, T.C., Wahlby, A. and Jones, T.A. (2004) The Uppsala Electron-Density Server. *Acta Crystallogr D Biol Crystallogr*, **60**, 2240-2249.

2. Zheng, H., Chruszcz, M., Lasota, P., Lebioda, L. and Minor, W. (2008) Data mining of metal ion environments present in protein structures. *J Inorg Biochem*, **102**, 1765-1776

3. Dudev, T., Cowan, J.A. and Lim, C. (1999) Competitive Binding in Magnesium Coordination Chemistry: Water versus Ligands of Biological Interest. *J. Am. Chem. Soc.*, **121**, 7665–7673.

4. Dudev, T. and Lim, C. (2004) Monodentate versus Bidentate Carboxylate Binding in Magnesium and Calcium Proteins: What Are the Basic Principles? *J. Phys. Chem. B*, **108**, 4546–4557.
