# Supplementary material for: How Cations Can Assist DNase I in DNA Binding and Hydrolysis
Source: PLoS Comput Biol. 2010 Nov 18;6(11):e1001000. doi: 10.1371/journal.pcbi.1001000 (PMC2987838; doi:10.1371/journal.pcbi.1001000)
Supplement: Table S1 — Ion binding sites predicted via the CHED server. This Table is related to Figure 1. The CHED server, available on line (http://ligin.weizmann.ac.il/~lpgerzon/mbs4), refers to Babor, M., Gerzon, S., Raveh, B., Sobolev, V. and Edelman, M. (2008) Prediction of transition metal-binding sites from apo protein structures. Proteins, 70, 208-217. N.O. stands for not observed. X marks the binding sites that were identified. a Both subsites IVa and IVb are identified as a single binding site. (0.04 MB DOC) [file pcbi.1001000.s002.doc]

**Table S1 :** ion binding sites predicted *via* the CHED server

This Table is related to Figure 1. The CHED server, available on line (<http://ligin.weizmann.ac.il/~lpgerzon/mbs4>), refers to Babor, M., Gerzon, S., Raveh, B., Sobolev, V. and Edelman, M. (2008) Prediction of transition metal-binding sites from *apo* protein structures. *Proteins*, **70**, 208-217.

N.O. stands for not observed. X marks the binding sites that were identified. ^a^ Both subsites IVa and IVb are identified as a single binding site.

| **PDB-ID** | **Resolution** | **Chain** | **Site I** | **Site II** | **Site III** | **Site IVa** | **Site IVb** |
| --- | --- | --- | --- | --- | --- | --- | --- |
| 3DNI | 2.00 Å | A | N.O. | X | X | X | X |
| 1ATN | 2.80 Å | D | N.O. | X | N.O. | X ^a^ | X ^a^ |
| 2D1K | 2.50 Å | B | N.O. | X | N.O. | X ^a^ | X ^a^ |
| 2A40 | 1.80 Å | B | N.O. | N.O. | N.O. | X ^a^ | X ^a^ |
| 2A40 | 1.80 Å | E | N.O. | X | N.O. | X ^a^ | X ^a^ |
| 2A42 | 1.85 Å | B | N.O. | N.O. | N.O. | X ^a^ | X ^a^ |
| 2A3Z | 2.08 Å | B | N.O. | X | N.O. | X ^a^ | X ^a^ |
| 2A41 | 2.60 Å | B | N.O. | X | N.O. | X ^a^ | X ^a^ |
| 2DNJ | 2.00 Å | A | N.O. | X | N.O. | X ^a^ | X ^a^ |
| 1DNK | 2.30 Å | A | N.O. | N.O. | N.O. | X ^a^ | X ^a^ |
