# Supplementary material for: How Cations Can Assist DNase I in DNA Binding and Hydrolysis
Source: PLoS Comput Biol. 2010 Nov 18;6(11):e1001000. doi: 10.1371/journal.pcbi.1001000 (PMC2987838; doi:10.1371/journal.pcbi.1001000)
Supplement: Table S2 — Thirty non-redundant DNase I sequences from various species. This Table is related to Table 5. The sequences homologous to bpDNase I correspond to precursors or mature proteins attributed to the DNase I family. The protein lengths are those of the original selected sequences. (0.05 MB DOC) [file pcbi.1001000.s003.doc]

**Table S2 :** 30 non-redundant DNase I sequences from various species

This Table is related to Table 5. The sequences homologous to bpDNase I correspond to precursors or mature proteins attributed to the DNase I family. The protein lengths are those of the original selected sequences.

| NCBI sequence identifier | Percentage of identity  with bpDNase I | Species | Protein length  (aa) |
| --- | --- | --- | --- |
| gi|118922 | 93 | Sheep | 260 |
| gi|47522808 | 80 | Sus scrofa | 284 |
| gi|126723112 | 79 | Equus caballus | 282 |
| gi|161016801 | 78 | Mus musculus | 284 |
| gi|114660657 | 78 | Pan troglodytes | 282 |
| gi|6978769 | 76 | Rattus norvegicus | 284 |
| gi|1197173 | 78 | Homo sapiens | 282 |
| gi|130500075 | 80 | Oryctolagus cuniculus | 281 |
| gi|109127402 | 79 | Macaca mulatta | 282 |
| gi|50950149 | 78 | Canis lupus familiaris | 284 |
| gi|224069926 | 60 | Taeniopygia guttata | 284 |
| gi|46395493 | 61 | Gallus gallus | 282 |
| gi|15823704 | 56 | Elaphe climacophora | 282 |
| gi|21388714 | 57 | Cyprinus carpio | 279 |
| gi|50540416 | 56 | Danio rerio | 278 |
| gi|18147095 | 55 | Gloydius halys | 282 |
| gi|56342183 | 55 | Heterodontus japonicus | 282 |
| gi|57157231 | 55 | Anguilla japonica | 282 |
| gi|2462635 | 54 | Oreochromis mossambicus | 284 |
| gi|6647483 | 54 | OREMO | 284 |
| gi|57157233 | 54 | Pagrus major | 281 |
| gi|56342181 | 51 | Triakis scyllium | 281 |
| gi|225706090 | 51 | Osmerus mordax | 287 |
| gi|14861058 | 48 | Rana catesbeiana | 353 |
| gi|14861062 | 45 | Bufo japonicus | 353 |
| gi|213626805 | 44 | Xenopus laevis | 350 |
| gi|229367158 | 48 | Anoplopoma fimbria | 273 |
| gi|14861060 | 43 | Cynops pyrrhogaster | 354 |
| gi|198424184 | 46 | Ciona intestinalis | 271 |
| gi|32475769 | 20 | Rhodopirellula baltica | 413 |
