# Supplementary material for: How Cations Can Assist DNase I in DNA Binding and Hydrolysis
Source: PLoS Comput Biol. 2010 Nov 18;6(11):e1001000. doi: 10.1371/journal.pcbi.1001000 (PMC2987838; doi:10.1371/journal.pcbi.1001000)
Supplement: Table S3 — Sixteen non-redundant DNase I-like 1 sequences from various species. This Table is related to Table 5. The sequences homologous to bpDNase I correspond to precursors or mature proteins attributed to the DNase I-like I family. The protein lengths are those of the original selected sequences. (0.04 MB DOC) [file pcbi.1001000.s004.doc]

**Table S3 :** 16 non-redundant DNase I-like 1 sequences from various species

This Table is related to Table 5. The sequences homologous to bpDNase I correspond to precursors or mature proteins attributed to the DNase I-like I family. The protein lengths are those of the original selected sequences.

| NCBI sequence identifier | Percentage of identity  with bpDNase I | Species | Protein length  (aa) |
| --- | --- | --- | --- |
| gi|151553571 | 43 | Bos taurus | 316 |
| gi|84874692 | 42 | Sus scrofa | 315 |
| gi|148697886 | 39 | Mus musculus | 288 |
| gi|84662732 | 42 | Rattus norvegicus | 312 |
| gi|5803007 | 41 | Homo sapiens | 302 |
| gi|91206583 | 43 | CRIGR | 304 |
| gi|160213460 | 42 | Papio anubis | 302 |
| gi|91206582 | 42 | CERAE | 302 |
| gi|170649635 | 41 | Callicebus moloch | 302 |
| gi|167045821 | 42 | Callithrix jacchus | 302 |
| gi|269120882 | 44 | Sebaldella termitidis | 252 |
| gi|217418274 | 44 | Oryctolagus cuniculus | 304 |
| gi|184185502 | 43 | Rhinolophus ferrumequinum | 314 |
| gi|74008844 | 43 | Canis familiaris | 295 |
| gi|194228453 | 43 | Equus caballus | 305 |
| gi|226955352 | 42 | Dasypus novemcinctus | 305 |
