# Supplementary material for: How Cations Can Assist DNase I in DNA Binding and Hydrolysis
Source: PLoS Comput Biol. 2010 Nov 18;6(11):e1001000. doi: 10.1371/journal.pcbi.1001000 (PMC2987838; doi:10.1371/journal.pcbi.1001000)
Supplement: Table S4 — Eleven non-redundant DNase I-like 2 sequences from various species. This Table is related to Table 5. The sequences homologous to bpDNase I correspond to precursors or mature proteins attributed to the DNase I-like 2 family. The protein lengths are those of the original selected sequences. (0.03 MB DOC) [file pcbi.1001000.s005.doc]

**Table S4 :** 11 non-redundant DNase I-like 2 sequences from various species

This Table is related to Table 5. The sequences homologous to bpDNase I correspond to precursors or mature proteins attributed to the DNase I-like 2 family. The protein lengths are those of the original selected sequences.

| NCBI sequence identifier | Percentage of identity  with bpDNase I | Species | Protein length  (aa) |
| --- | --- | --- | --- |
| gi|224069930 | 57 | Taeniopygia guttata | 330 |
| gi|119120921 | 53 | Monodelphis domestica | 288 |
| gi|157279947 | 53 | Bos taurus | 278 |
| gi|125381322 | 58 | Gallus gallus | 237 |
| gi|194219348 | 54 | Equus caballus | 302 |
| gi|109487831 | 52 | Rattus norvegicus | 278 |
| gi|148539953 | 52 | Mus musculus | 278 |
| gi|4503347 | 53 | Homo sapiens | 299 |
| gi|109127257 | 44 | Macaca mulatta | 253 |
| gi|73959491 | 45 | Canis familiaris | 218 |
| gi|260775755 | 35 | Vibrio coralliilyticus | 294 |
