# Supplementary material for: How Cations Can Assist DNase I in DNA Binding and Hydrolysis
Source: PLoS Comput Biol. 2010 Nov 18;6(11):e1001000. doi: 10.1371/journal.pcbi.1001000 (PMC2987838; doi:10.1371/journal.pcbi.1001000)
Supplement: Table S5 — Seventeen non-redundant DNase I-like 3 sequences from various species. This Table is related to Table 5. The sequences homologous to bpDNase I correspond to precursors or mature proteins attributed to the DNase I-like 3 family. The protein lengths are those of the original selected sequences. (0.04 MB DOC) [file pcbi.1001000.s006.doc]

**Table S5 :** 17 non-redundant DNase I-like 3 sequences from various species

This Table is related to Table 5. The sequences homologous to bpDNase I correspond to precursors or mature proteins attributed to the DNase I-like 3 family. The protein lengths are those of the original selected sequences.

| NCBI sequence identifier | Percentage of identity  with bpDNase I | Species genome | Protein length  (aa) |
| --- | --- | --- | --- |
| gi|77736625 | 52 | Gallus gallus | 307 |
| gi|51261555 | 50 | Xenopus (Silurana) tropicalis | 295 |
| gi|225708354 | 49 | Osmerus mordax | 318 |
| gi|223647596 | 48 | Salmo salar | 298 |
| gi|154757658 | 50 | Bos taurus | 295 |
| gi|3236320 | 48 | Homo sapiens | 305 |
| gi|109037932 | 48 | Macaca mulatta | 305 |
| gi|16758784 | 48 | Rattus norvegicus | 310 |
| gi|84490435 | 48 | Mus musculus | 310 |
| gi|225714724 | 45 | Esox lucius | 266 |
| gi|225707660 | 45 | Osmerus mordax | 268 |
| gi|33604227 | 45 | Danio rerio | 315 |
| gi|229365908 | 43 | Anoplopoma fimbria | 324 |
| gi|259089125 | 43 | Oncorhynchus mykiss | 306 |
| gi|87307062 | 27 | Blastopirellula marina | 328 |
| gi|149175989 | 25 | Planctomyces maris | 343 |
| gi|32472155 | 22 | Rhodopirellula baltica | 341 |
